# Dynamic evolution of the sofosbuvir-associated variant A1343V in HEV-infected patients under concomitant sofosbuvir-ribavirin treatment

**DOI:** 10.1016/j.jhepr.2023.100989

**Published:** 2024-01-03

**Authors:** André Gömer, Katja Dinkelborg, Mara Klöhn, Michelle Jagst, Michael Hermann Wißing, Nicola Frericks, Pia Nörenberg, Patrick Behrendt, Markus Cornberg, Heiner Wedemeyer, Eike Steinmann, Benjamin Maasoumy, Daniel Todt

**Affiliations:** 1 Department of Molecular and Medical Virology, Ruhr University Bochum, Bochum, Germany; 2 Department of Gastroenterology, Hepatology and Endocrinology, Hannover Medical School, Germany; 3 TWINCORE, Centre for Experimental and Clinical Infection Research, a Joint Venture between the Medical School Hannover (MHH) and the Helmholtz Centre for Infection Research (HZI), Hannover, Germany; 4 German Center for Infectious Disease Research (DZIF); Partner Sites Hannover-Braunschweig, Germany; 5 Institute of Virology, University of Veterinary Medicine Hannover, Hannover, Germany; 6 Excellence Cluster 2155 RESIST, Hannover Medical School, Hannover, Germany; 7 German Centre for Infection Research (DZIF), External Partner Site, Bochum, Germany; 8 European Virus Bioinformatics Center (EVBC), Jena, Germany

**Keywords:** Viral RNA-directed RNA polymerase inhibitors, high-throughput sequencing, resistance profiling, intra-host evolution

## Abstract

**Background & Aims:**

In the absence of a hepatitis E virus (HEV)-specific antiviral treatment, sofosbuvir has recently been shown to have antiviral activity against HEV *in vivo*. However, a variant, A1343V, that is strongly associated with viral relapse impedes treatment success. In this study, we investigated the occurrence of variants during sofosbuvir and ribavirin treatment *in vivo* and assessed the sensitivity of resistance-associated variants to concurrent treatment in cell culture.

**Methods:**

Two patients with chronic HEV infection that did not clear infection under ribavirin treatment were subsequently treated with a combination of sofosbuvir and ribavirin. We determined response to treatment by measuring liver enzymes and viral load in blood and stool. Moreover, we analyzed viral evolution using polymerase-targeted high-throughput sequencing and assessed replication fitness of resistance-associated variants using a HEV replicon system.

**Results:**

Combination treatment was successful in decreasing viral load towards the limit of quantification. However, during treatment sustained virological response was not achieved. Variants associated with sofosbuvir or ribavirin treatment emerged during treatment, including A1343V and G1634R. Moreover, A1343V, as a single or double mutation with G1634R, was associated with sofosbuvir resistance during concomitant treatment *in vitro*.

**Conclusions:**

These results highlight the importance of variant profiling during antiviral treatment of patients with chronic infection. Understanding how intra-host viral evolution impedes treatment success will help guide the design of next-generation antivirals.

**Impact and implications:**

The lack of hepatitis E virus (HEV)-specific antivirals to treat chronic infection remains a serious health burden. Although ribavirin, interferon and sofosbuvir have been reported as anti-HEV drugs, not all patients are eligible for treatment or clear infection, since resistant-associated variants can rapidly emerge. In this study, we analyzed the efficacy of sofosbuvir and ribavirin combination treatment in terms of HEV suppression, the emergence of resistance-associated variants and their ability to escape treatment inhibition *in vitro*. Our results provide novel insights into evolutionary dynamics of HEV during treatment and thus will help guide the design of next-generation antivirals.

## Highlights

• Sofosbuvir and ribavirin combination therapy efficiently decreased viral RNA in blood and stool in patients with chronic HEV infection.
• Variants that are associated with antiviral resistance emerged during treatment.
• High-dose combination treatment is efficient in repressing viral replication of single and multi-variant clones *in vitro.*

## Graphical abstract

**Figure:**
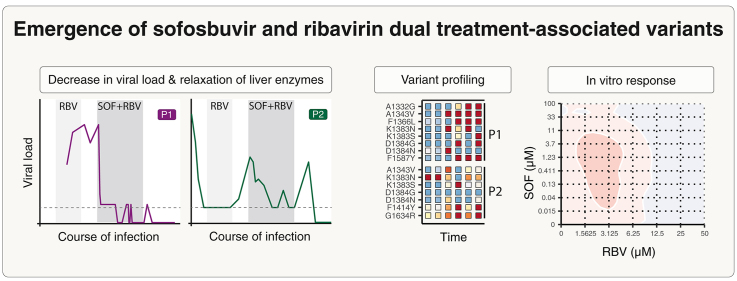

## Introduction

Hepatitis E virus (HEV) is a single-stranded RNA virus that causes an estimated 20 million acute self-limiting infections annually, making it one of the most common causes of viral hepatitis.^1^ In immunocompromised patients, HEV frequently persists and develops into a chronic infection (CHE).^1^ Currently, there is no specific treatment for symptomatic HEV infection, leaving over 3.3 million patients at risk of severe liver disease.[2], [3], [4] The only treatment options for these patients are the off-label use of ribavirin (RBV), which is associated with a sustained virological response (SVR) of ∼80%, or interferon-based regimens, which are associated with severe side effects.^5^^,^^6^ In addition, patients with renal insufficiency, those at risk for graft rejection or pregnant women are not eligible for either treatment, highlighting the urgent need for new anti-HEV drugs.^7^

Sofosbuvir (SOF), a hepatitis C virus polymerase inhibitor, has been evaluated for the treatment of patients with CHE.[8], [9], [10], [11], [12], [13], [14], [15], [16], [17], [18], [19] Although the initial response, as measured by decreasing RNA levels, was promising in multiple case reports, SVR was often not achieved, as viral RNA levels rebounded during treatment. Recently, we have demonstrated that the emergence of a substitution from alanine to valine at position 1343 in the viral polymerase was strongly associated with viral relapse. This variant led to a strong decrease in SOF sensitivity *in vitro*, but had no effect on RBV susceptibility.^20^ Thus, the combination of RBV and SOF could suppress the effect of resistance-associated mutations against both drugs.

Here, we investigate the emergence of SOF-associated variants during concomitant treatment with SOF and RBV in two patients with CHE. In addition, we analyzed whether the combination therapy could overcome the effect of resistance-associated variants.

## Materials and methods

### Patients

Both patients were treated at Hannover Medical School and provided written informed consent before enrolment in the study (ethical approval: 8314_BO_K_2019). Blood and stool samples were taken during routine appointments. There were no additional procedures performed with the patients for this study. HEV RNA levels and all other laboratory parameters were measured by the central laboratory of Hannover Medical School.

### *In vitro* assay

We examined the sensitivity of Kernow-C1 p6 (wild-type [WT] control, HEV-3c) and the A1343V, G1634R, A1343V/G1634R mutant (in Kernow-C1 p6 background) to SOF (Medchemexpress (HY-15005)) and RBV (Sigma Aldrich (R9644)) by electroporating *in vitro-*transcribed RNA into HepG2 cells. Both drugs were dissolved in DMSO. Supernatants were collected 72 h after treatment, and Gaussia luciferase activity was measured using a Centro XS3 LB 960 luminometer (Berthold Technologies). In parallel, cell viability was measured using the MTT (3-(4,5-dimethylthiazol-2-yl)-2,5-diphenyltetrazolium bromide) assay.^20^

### Amplicon generation & sequencing

Total RNA from 200 μl serum was extracted using the Cobas AmpliPrep total nucleic acid isolation kit (Roche, Basel, Switzerland). Complementary DNA (cDNA) and amplicons were prepared according to Todt *et al.*.^21^ Next-generation sequencing libraries were prepared using the Nextera XT DNA Library Preparation Kit (Illumina, San Diego, CA, USA). Libraries were sequenced at 2 × 250 bp on a MiSeq using a paired-end sequencing protocol.

### Variant analysis

Data analysis was based on the pipeline described in.^22^ Raw data were trimmed using Trimmomatic 0.39 and quality checked using FastQC (https://www.bioinformatics.babraham.ac.uk/projects/fastqc/). Baseline consensus sequences were called using SAM2Consesus v2.0 (https://github.com/vbsreenu/Sam2Consensus). Each patient's serum consensus sequence at baseline was used as a reference for subsequent mapping and variant analysis with Diversitools (https://github.com/josephhughes/DiversiTools),vNvS tools (https://github.com/rjorton/vnvs) and cliqueSNV. Data were visualized with custom R scripts using the following packages: Tidyverse, ggpubr, and Patchwork.

## Results

**Fig. 1:**
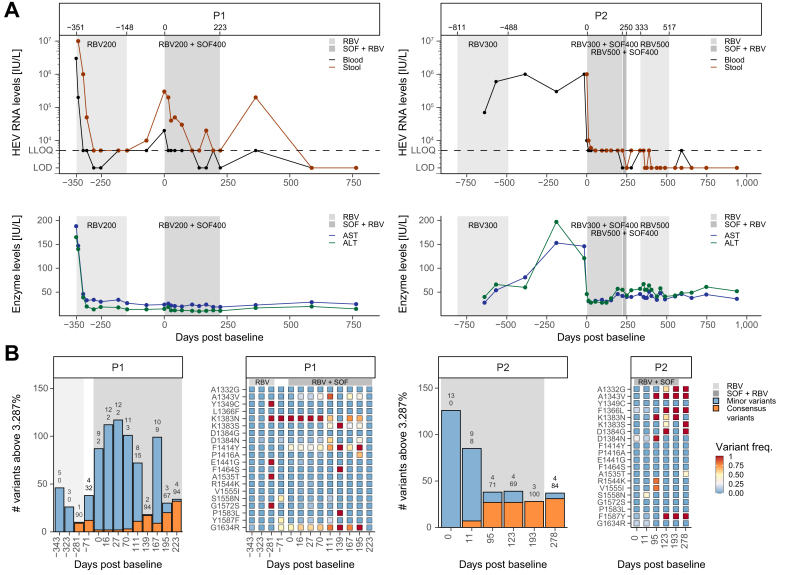
Course of treatment with RBV and RBV-SOF in patients with CHE. (A) HEV RNA levels (UI/L) from stool (brown) and blood (black). The lower limit of quantification is set at 5,000 UI/L. The lower panel shows liver enzyme levels: AST (green) and ALT (blue). (B) HEV variant analysis from stool samples. Nucleotide variants, defined as non-reference nucleotide above 3.287 percent (gray) and consensus variants above 50 percent (orange). The right panel shows amino acid frequencies of substitutions that reached the consensus level during the treatment period. ALT, alanine aminotransferase; AST, aspartate aminotransferase; CHE, chronic HEV infection; HEV, hepatitis E virus; RBV, ribavirin; SOF, sofosbuvir.

Herein, we present data on two patients with CHE (genotype 3c, Fig. S1) receiving combinational antiviral therapy with ribavirin and sofosbuvir. Both patients were solid organ transplant recipients under immunosuppressive therapy, most likely causing the chronicity of the infection (patient 1: kidney transplantation, prednisolone, everolimus and tacrolimus; patient 2: lung transplantation, prednisolone and tacrolimus; Table S1). A reduction of the immunosuppressive regimen was considered but not feasible due to the risk of organ rejection. Additionally, both patients had failed earlier RBV treatment. Patient 1 had received 7 months of RBV therapy in which HEV RNA levels showed a profound decrease. However, the patient relapsed after therapy (Fig. 1A). Interestingly, liver enzymes decreased during RBV therapy and stayed within normal levels. Additionally, the patient did not show signs of increased liver fibrosis (Table S1). Patient 2 had received RBV twice for 3 and 13 months. Although HEV RNA levels decreased slightly, they remained above the limit of detection (Fig. 1A). In addition, patient 2 had elevated alanine aminotransferase and aspartate aminotransferase levels, and indications of non-invasive liver fibrosis (FibroScan), although sonograms showed no signs of advanced cirrhosis. At this stage, both patients were treated with a combination of RBV and SOF. Despite a slight reduction in hemoglobin levels (≈0.5 g/dl), no other side effects were apparent in either patient.

Patient 1 received 200 mg RBV daily in combination with 400 mg SOF daily. HEV RNA levels declined from 3 × 10^5^ IU/L in stool (blood: 2 × 10^4^ IU/L) to below the lower limit of quantification (LLOQ) at day 42 of treatment; however, they also peaked back at 2 × 10^4^ IU/L after 167 days. At the end of treatment, HEV RNA was below the LLOQ in stool and undetectable in blood; however, 4 months post treatment, viral RNA was detectable in stool (2 × 10^5^ IU/L). Thereafter, it was undetectable for 12 and 17 months. Patient 1 passed away 24 months after treatment due to an HEV-unrelated disease.

In patient 2, HEV RNA levels in blood (pre-treatment: 1 × 10^6^ IU/L) and stool (5 × 10^3^ IU/L) rapidly decreased to below the LLOQ during treatment with 300 mg RBV (alternating 200 mg and 400 mg daily) and 400 mg SOF daily. Although HEV RNA was undetectable in blood after 223 days of treatment, HEV RNA remained detectable in stool. At the same time, co-treatment caused a reduction in liver enzyme levels. In an attempt to clear HEV RNA completely, the RBV dosage was increased to alternating 400 mg and 600 mg daily, which led to negative PCR results in both specimens at day 250 after treatment initiation. One month post treatment, HEV RNA levels relapsed in stool below the LLOQ. This led us to initiate another 6 months of 500 mg daily RBV therapy after which HEV RNA has not been detected for 1.5 years.

To identify variants that may impede treatment efficacy, we performed high-throughput sequencing, targeting the HEV polymerase, on stool samples from these patients (Fig. 1B). We compared variants for each of the patients with the first time point available for high-throughput sequencing (Fig. S2).

For patient 1, viral populations were more heterogenous early during SOF-RBV treatment (9% minor variants, above the error-threshold of 3.287 percent). As treatment progressed, the number of minor variants decreased, while the number of consensus variants (above 50 percent) increased. Similarly, patient 2 had a virus population with higher diversity at treatment initiation (13% minor variants) and fewer minor variants but more consensus variants at the end of treatment. Consistent with this, patient 2 showed a strong accumulation of substitutions during treatment, including A1343V. We observed similar variants in patient 1, including the A1343V variant, which became transiently dominant during treatment, and the G1634R. This pattern of evolution was similar when using haplotype reconstruction, which indicated that A1343V and G1634R were not present on the same genome in patient 2 (Fig. S3).

**Fig. 2:**
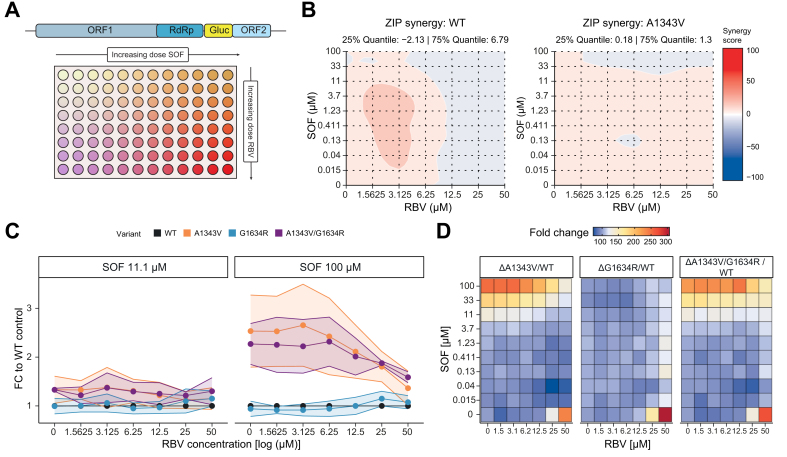
*In vitro* analysis of simultaneous treatment with RBV-SOF on A1343V. (A) Experimental setup. (B) Synergy analysis of A1343V and WT control replicon data generated by titration of RBV-SOF. Synergy values between 10 and -10 indicate additive effects, above 10 synergistic effects and below -10 antagonistic effects. (C) Replication capacity of A1343V, G1634R, A1343V/G1634R and WT control during treatment with 11 μM (left) or 100 μM (right) SOF and simultaneous titration of RBV (from 0–50 μM). (D) Normalized replication of the variants compared to WT control: Red indicates stronger replication and blue indicates lower replication than WT. FC, fold-change; RBV, ribavirin; SOF, sofosbuvir; WT, wild-type; ZIP, zero interaction potency.

Next, we investigated whether the combination of SOF and RBV can overcome the resistance-associated phenotype of A1343V or G1634R in tissue culture. To this end, we titrated both drugs in a matrix-type combinatorial replicon assay to determine whether they act synergistically (Fig. 2A). Synergy analysis indicated additive effects of the two drugs (zero interaction potency score between 10 to -10). While in the WT control the effect became slightly weaker, it remained similar for the variants (Fig. 2B). Importantly, higher concentrations of both drugs appear to have the strongest effect on viral replication: At 100 μM SOF, the A1343V and the A1343V/G1634R variants were about 2.4-times less efficiently inhibited than the WT control (Fig. 2C,D). At the same time, the G1634R variant did not lead to increased resistance (0.99-fold). However, the difference in replication between WT and the variants becomes smaller when the RBV concentration increases and all variants showed a low replication capacity at maximum concentrations of both drugs (fold-change to WT: A1343V 1.3, G1634R 1.1, A1343V/G1634R 1.5). At the same time, cytotoxic effects (as determined by the MTT assay) remained modest (Fig. S4C, D).

In summary, resistant-associated variants emerged in patients with CHE treated with RBV and SOF. The effect of the A1343V and A1343V/G1634R double mutant, however, could be mitigated with high doses of RBV and SOF *in vitro*.

## Discussion

Herein, we describe the course of chronic HEV infection and the emergence of resistance-associated variants in two organ transplant recipients treated with SOF and RBV. These patients had previously been treated with RBV monotherapy, which did not result in a sustained virologic response. In addition, we demonstrated that the combination of SOF and RBV could overcome the effect of resistance-associated mutations *in vitro*.

Overall, treatment was well tolerated by both patients. We closely monitored HEV RNA levels in stool and blood during concurrent treatment to ensure that, if clearance is possible, all virus is removed. This may also be an important monitoring procedure, as HEV often remains longer in stool, possibly indicating residual viral reservoirs.^11^^,^^12^ Thus, stool sequencing may increase the detection window and increase the sensitivity of detection of minor variants. Indeed, at the end of treatment, both patients were able to clear HEV in plasma but not in stool samples.

At first glance, the primary outcome (no SVR) may not be surprising, as this has also been observed in several other studies.[9], [10], [11], [12], [13], [14], [15], [16]^,^^18^ Most of these studies also used a combination of SOF and RBV for treatment, and often SOF was added to an ongoing RBV treatment. As we have previously shown, highly diverse virus populations may already harbor resistance-associated variants at low frequency – such as the A1343V. RBV may thus promote virus heterogeneity which in turn promotes the emergence of variants. However, it is interesting to note that viral relapse was weaker and delayed in these two patients, as indicated by viral loads near the limit of detection throughout treatment, especially compared to SOF monotherapy, where viral relapse occurred between week 4 and 12 after treatment initiation.

Recently, we have investigated the impact of emerging variants and HEV relapse during SOF monotherapy in nine patients.^20^ Indeed, the pattern of intra-host evolution was similar, including changes in viral diversity, from highly diverse at treatment start to less diverse, but with a higher number of consensus changes. Moreover, similar variants occurred at high frequency, namely at position: 1383, 1384, 1587, and 1634. Of note, we have also detected variants that, to our knowledge, were not previously characterized: A1332G, F1366L, but have been detected post SOF and RBV treatment in a patient that did not clear CHE.^14^ Since our study had the clear limitation of only including two patients, future studies should include more patients to unravel evolutionary dynamics involved in treatment resistance and characterize emerging variants *in vitro*.

Recently, we found strong evidence that HEV relapse during SOF monotherapy is associated with a specific mutation, A1343V, in the HEV polymerase finger domain. This mutation occurred in eight of nine patients at the consensus level and was associated with a 5-fold decrease in SOF susceptibility *in vitro*.^20^ The same mutation, although at differing frequencies, occurred in the two patients presented in this study who were treated with SOF and RBV. To gain more insight into whether this mutation could also be a determinant of antiviral resistance, we examined the effect of the two drugs on A1343V, G1634R and the double mutant *in vitro*. Indeed, the resistance phenotype was strongly suppressed at high concentrations of both drugs for all variants, as indicated by strong inhibition of viral replication. It should be noted that, as reported in other studies, about 20% cytotoxicity was observed at high concentrations of both drugs, which may affect the read-out. However, to what degree this concentration can be translated into clinics remains open. In general, interpreting how a particular variant contributes to antiviral resistance in a complex population where multiple variants fluctuate at a given time during treatment remains difficult. In addition, amplifying from low viral load samples may lead to misrepresentation of the true diversity in a particular patient.

In conclusion, resistance-associated variants occurred in two patients treated with SOF and RBV. *In vitro* studies have shown that high doses of both drugs can abrogate the fitness advantage of resistance-associated variants. Since there are currently only few reports available, future studies should systematically investigate the effects of concurrent treatment and drug doses, while monitoring HEV diversity.

## Financial support

D.T. was supported by grants from the German Ministry of Education and Research (BMBF, project VirBio; 01KI2106). E.S. was supported by grants of the German Federal Ministry of Health (ZMVI1-2518FSB705), from the German Research Foundation (DFG, grant number: 398066876-GRK 2485/1) and the German Ministry of Education and Research (BMBF, project SILVIR: 16GW0202). M.C., H.W., P.B. and E.S. were funded by a grant from the German Centre for Infection Research (DZIF). H.W. and E.S. were funded by the German Ministry of Education and Research (HepEDiaSeq 01EK2106A/B). All authors had access to the study data and reviewed and approved the final manuscript.

## Authors’ contributions

Contributed to the conception and design of the study: AG, KD, ES, BM, DT. Provided administrative, study supervision, and obtained funding: PB. HW, ES, BM, DT. Provided human specimens: KD, PN, PB, HW, BM. Performed experiments and substantially contributed to the acquisition of data and its analysis: AG, KD, MK, MJ, MHW, NF, ES, BM, DT. Interpretation of data: AG, KD, MK, MJ, MHW, NF, PB, HW, ES, BM, DT. Drafted the manuscript: AG, KD, ES, DT. Revised the manuscript critically for important intellectual content: All authors.

## Data availability statement

Associated NGS data is available at (https://doi.org/10.17632/2bcrjwf2ph.1).

## Conflicts of interest

H.W. received grants, consulting feed and honoraria from Abbvie, Aligos, Altimmune, Biotest, BMS, BTG, Dicerna, Enanta, Gilead, Janssen, Merck/MSD, MYR GmbH, Roche, Vri Biotechnology. In addition H.W. served as clinical trial investigator for Abbvie, Altimmune, BMS, Gilead, Jansen, Merck/MSD, MYR GmbH, Novartis, Vri Biotechnology. M.C. received consulting fees, honoraria or travel support by: Abbvie, AiCuris, Gilead, GlaxoSmithKline, Janssen–Cilag, MSD Sharp & Dohme, Spring Bank Pharmaceuticals, Swedish Orphan Biovitrum AB (SOBI) and Falk. Moreover, M.C. is on the board of the German Liver Foundation (GASL: 2017–2020). P.B. received funding from the German Centre for infection research (DZIF). B.M. received grants, consulting feed, honoraria and travel support from: Roche Diagnostics, Fujirebio, EWIMED, AbbVie, Luvos, Norgine and Gilead. In addition, B.M. own Biotech stocks.

Please refer to the accompanying ICMJE disclosure forms for further details.
