# Supplementary material for: Dynamic evolution of the sofosbuvir-associated variant A1343V in HEV-infected patients under concomitant sofosbuvir-ribavirin treatment
Source: JHEP Rep. 2024 Jan 3;6(3):100989. doi: 10.1016/j.jhepr.2023.100989 (PMC10906529; doi:10.1016/j.jhepr.2023.100989)
Supplement: Multimedia component 1 [file mmc1.pdf]

Dynamic evolution of the sofosbuvir-associated variant A1343V in HEV-infected patients under concomitant sofosbuvir-ribavirin treatment

André Gömer, Katja Dinkelborg, Mara Klöhn, Michelle Jagst, Michael Hermann Wißing, Nicola Frericks, Pia Nörenberg, Patrick Behrendt, Markus Cornberg, Heiner Wedemeyer, Eike Steinmann, Benjamin Maasoumy, Daniel Todt

Table of contents

Fig. S1……………………………………………………………………………………2

Fig. S2……………………………………………………………………………………3

Fig. S3……………………………………………………………………………………4

Fig. S4……………………………………………………………………………………5

Table S1………………………………………………………………………………....6

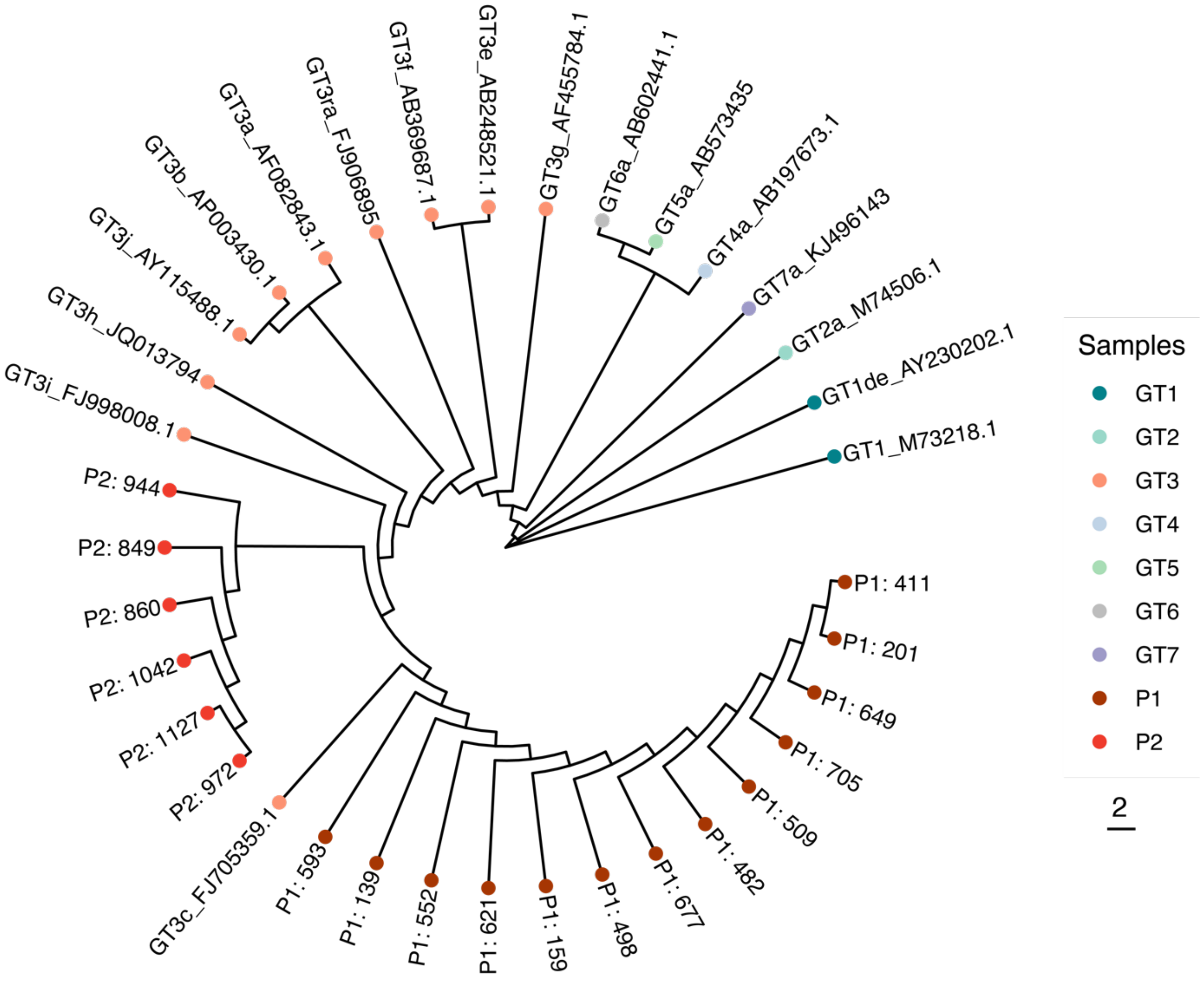

Fig. S1: Genotype classification. Maximum likelihood tree including consensus sequences from samples from patient 1 (P1, brown) and patient 2 (P2, orange). Reference sequence for HEV GT1 – GT7 were added. This tree was build using IQtree with 1000 boostraps.

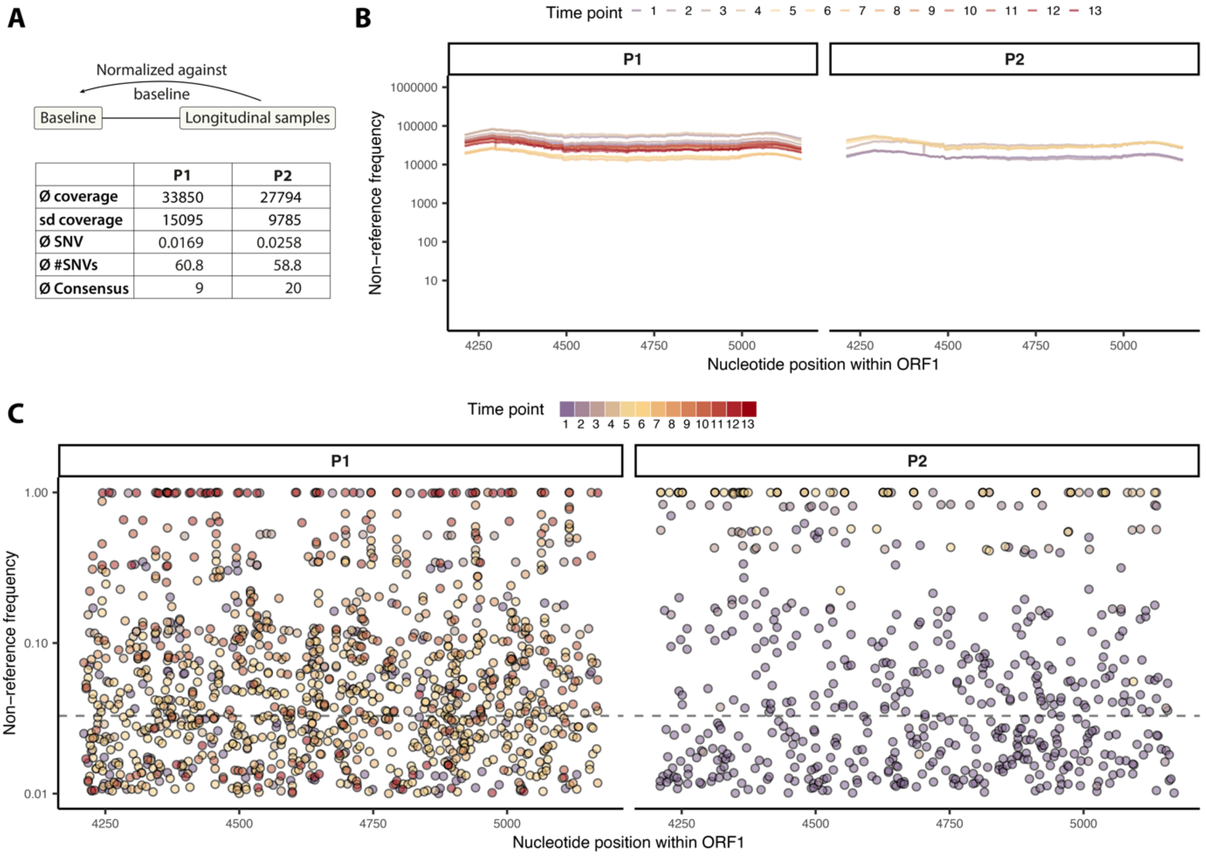

Fig. S2: Quality assessment and statistics of virus population analysis. (A) Illustration for consensus calling and analysis of samples against baseline. The table shows statistics from viral NGS. (B) Coverage over the genome. Colors depict sample time points. (C) Nucleotide variants per time point and patient.

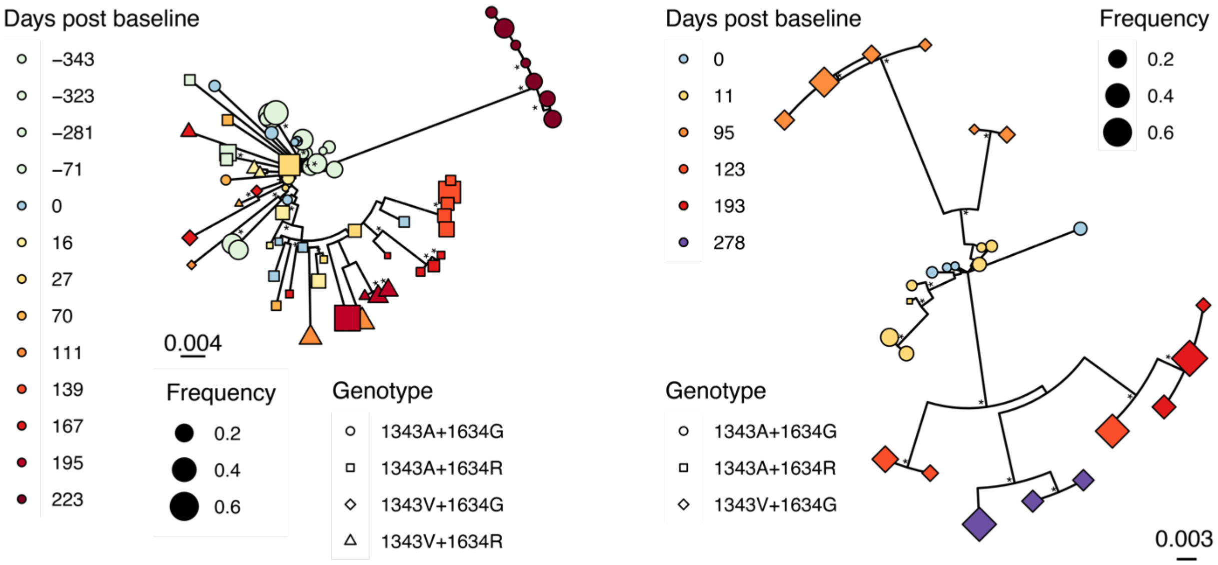

Fig. S3: Variant linkage analysis. CliqueSNV was used to construct haplotypes from NGS reads for phylogenetic analysis. Maximum likelihood tree was generated using IQtree (best model find option with 1000 bootstraps). Bootstrap support (>= 80%) indicated with as asterix. Haplotype frequency is indicated as dot size. Time point post baseline is color coded from early (blue colors) to later time points (red). The combination of A1343V and G1634R is indicated as tip shape.

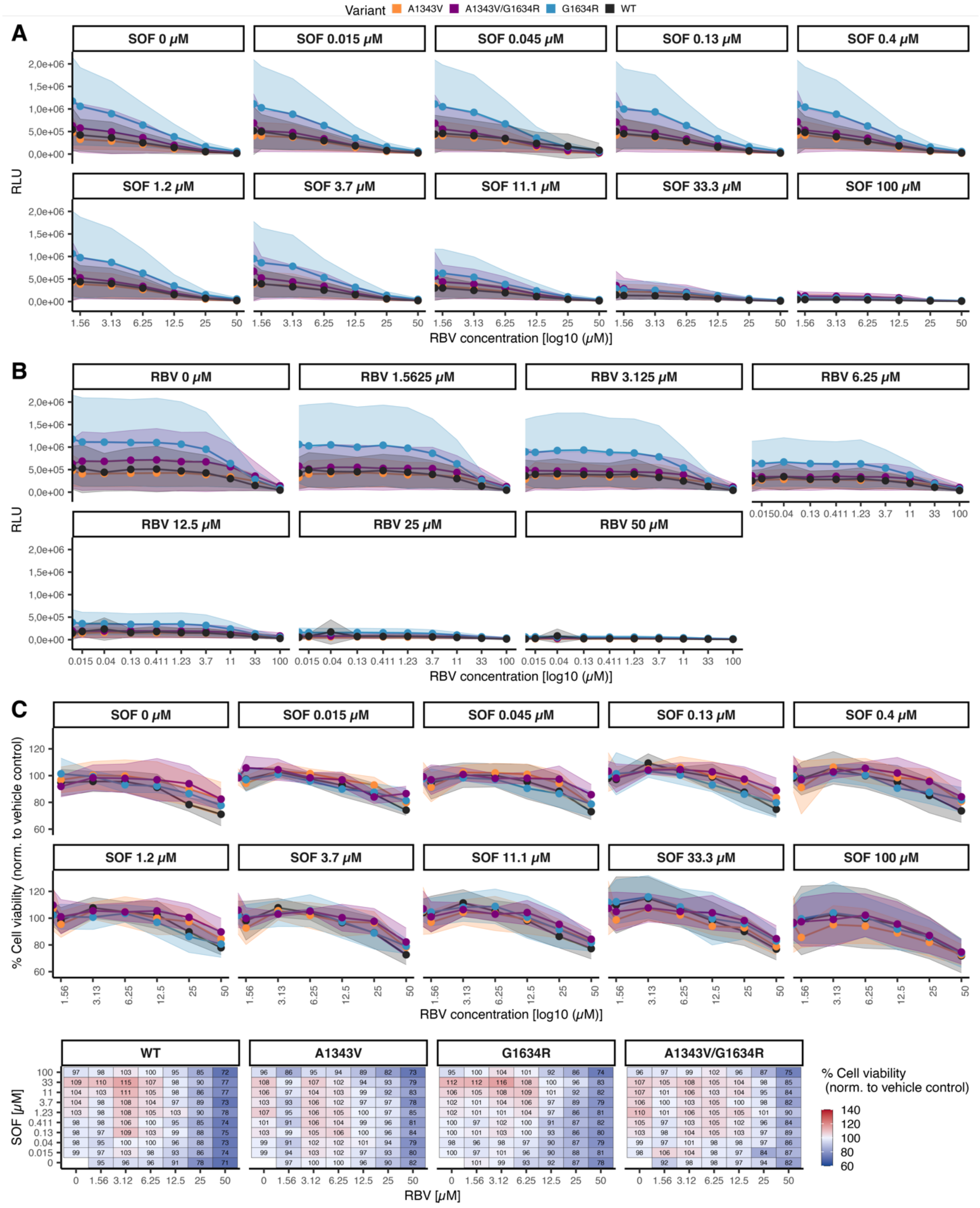

Fig. S4: Antiviral activity of SOF and RBV co-treatment. (A, B) Stable concentration of SOF (A) and RBV (B) against the other drug. WT control (Kernow C1 p6) in dark grey, A1343V (orange), G1634R (blue), A1343V/G1634R (purple). (C) Cytotoxicity assay (MTT) for all variants and titration of both drugs.

Table S1: baseline defined as start of RBV/SOF treatment

Patient	P2 (BL: 31.03.2020)	P1 (BL: 31.3.2020)
Age (years)	58	74
Sex	male	male
**HEV-RNA (IU/mL) blood**	1,000,000	20,000
**HEV-RNA (IU/mL) stool**	1,000,000	300,000
GPT (IU/L, norm ≤45)	121	15
GOT (IU/L, norm ≤35)	146	24
Bilirubin (µmol/L, norm 2-21)	12	5
Glomerular filtration rate ($ml/min/1.73m^2$, **norm** >90)	33	24
INR (norm 0.90-1.25)	0.94	1.78#
Platelets ($10^3/\mu l$, norm 160-370)	216	217
Fibroscan LSM/IQR (kPa)	43.4	5.2
FIB4 index	1.93	0.316
Duration of previous RBV therapy (months)	16 (1st: 3 months, 2nd: 13 months)	7
Mean dose of previous RBV therapy (mg)	350	200
**Response to previous RBV therapy**	NR	NR
Duration of RBV/SOF therapy (months)	8	8
Mean dose of RBV during RBV/SOF therapy (mg)	400	200
Immune suppressive condition	Lung Tx	Kidney Tx
Immunosuppressive regimen		
Cortisone	X	X
Tacrolimus	X	X
Everolimus		X

All values from baseline (start of RBV/SOF); GOT, glutamic oxaloacetic transaminase; GPT, glutamic pyruvic transaminase; LSM, liver stiffness measurement; APRI, aspartate transaminase-to-platelet ratio index; NR, non-response (breakthrough or never HEV RNA negative); Tx, transplantation; Tacrolimus, Calcineurin inhibitor; Everolimus, mTOR inhibitor.

#under Phenprocoumon therapy
