# Supplementary material for: Dynamic evolution of the sofosbuvir-associated variant A1343V in HEV-infected patients under concomitant sofosbuvir-ribavirin treatment
Source: JHEP Rep. 2024 Jan 3;6(3):100989. doi: 10.1016/j.jhepr.2023.100989 (PMC10906529; doi:10.1016/j.jhepr.2023.100989)
Supplement: Multimedia component 2 — : [file mmc2.docx]

**JHEP Reports**

**CTAT methods**

Tables for a “Complete, Transparent, Accurate and Timely account” (CTAT) are now mandatory for all revised submissions. The aim is to enhance the reproducibility of methods.

- Only include the parts relevant to your study
- Refer to the CTAT in the main text as ‘Supplementary CTAT Table’
- Do not add subheadings
- Add as many rows as needed to include all information
- Only include one item per row

**If the CTAT form is not relevant to your study, please outline the reasons why:**

|  |
| --- |

- 1. **Antibodies**

| **Name** | **Citation** | **Supplier** | **Cat no.** | **Clone no.** |
| --- | --- | --- | --- | --- |
|  |  |  |  |  |

- 1. **Cell lines**

| **Name** | **Citation** | **Supplier** | **Cat no.** | **Passage no.** | **Authentication test method** |
| --- | --- | --- | --- | --- | --- |
| HepG2 |  | ATCC | HB8065 | 20 – 30 | regular RNAseq validation |

- 1. **Organisms**

| **pName** | **Citation** | **Supplier** | **Strain** | **Sex** | **Age** | **Overall n number** |
| --- | --- | --- | --- | --- | --- | --- |
|  |  |  |  |  |  |  |

- 1. **Sequence based reagents**

| **Name** | **Sequence** | **Supplier** |
| --- | --- | --- |
| F-O-HEV_ORF1 | ACGCTYGTGGGYAGGTACGG | Merck |
| R-O-HEV_ORF1 | AGCAYGARGARCAGCAACAC | Merck |
| F-I-HEV_ORF1 | YTCTGAYGTCCGTGAGTCCC | Merck |
| R-I-HEV_ORF1 | TATGYACCARBCCRGGRCTA | Merck |
| R-HEV_ORF1_cDNA | AGGGGTTGGTTGGATGAATA | Merck |

- 1. **Biological samples**

| **Description** | **Source** | **Identifier** |
| --- | --- | --- |
| Patient sample 1 | Human serum & stool | P1 |
| Patient sample 2 | Human serum & stool | P2 |

- 1. **Deposited data**

| **Name of repository** | **Identifier** | **Link** |
| --- | --- | --- |
| Mendeley data |  | DOI: 10.17632/2bcrjwf2ph.1 |

- 1. **Software**

| **Software name** | **Manufacturer** | **Version** |
| --- | --- | --- |
| Trimmomatic | http://www.usadellab.org/cms/?page=trimmomatic | 0.39 |
| Tanoti | https://bioinformatics.cvr.ac.uk/software/tanoti/ |  |
| Samtools | http://htslib.org/ | 1.9 |
| Gatk4 | https://software.broadinstitute.org/gatk/ | 4.3.0.0 |
| SAM2CONSENSUS | https://github.com/vbsreenu/Sam2Consensus | 2.0 |
| CliqueSNV | https://github.com/vtsyvina/CliqueSNV | 2.0.3 |
| Diversitools | http://josephhughes.github.io/DiversiTools/ | 0.1 |
| Vnvs tools | https://github.com/rjorton/vnvs |  |
| R | https://cran.r-project.org | 4.3.1 |
| SynergyFinder | http://www.synergyfinder.org | 3.10.0 |
| Tidyverse | https://tidyverse.tidyverse.org | 2.0.0 |
| ggpubr | <https://cran.r-project.org/web/packages/ggpubr/index.html> | 0.6.0 |
| IQtree | <http://www.iqtree.org> | 2.0.3 |

- 1. **Other (*e.g*. drugs, proteins, vectors etc.)**

| **Drug** | **Source** |  |
| --- | --- | --- |
| Sofosbuvir | https://www.medchemexpress.com/PSI-7977.html |  |
| Ribavirin | https://www.sigmaaldrich.com/DE/de/product/sigma/r9644 |  |
| pBlueScript SK(+) encoding Kernow-C1 p6 gluc clone, HEV-3; GenBank accession no. JQ679013 | Kindly provided  by Suzanne U. Emerson |  |

- 1. **Please provide the details of the corresponding methods author for the manuscript:**

| Dr. Daniel Todt  Department of Molecular and Medical Virology  Ruhr-University Bochum  Universitätsstr. 150  44801 Bochum, Germany  Phone: +49 234 32 23189, Fax: +49 234 32 14352  Email: Daniel.todt@ruhr-uni-bochum.de |
| --- |

**2.0 Please confirm for randomised controlled trials all versions of the clinical protocol are included in the submission. These will be published online as supplementary information.**

|  |
| --- |
